# Intrafamily Transmission of Monkeypox Virus, Central African Republic, 2018

**DOI:** 10.3201/eid2508.190112

**Published:** 2019-08

**Authors:** Camille Besombes, Ella Gonofio, Xavier Konamna, Benjamin Selekon, Antoine Gessain, Nicolas Berthet, Jean-Claude Manuguerra, Arnaud Fontanet, Emmanuel Nakouné

**Affiliations:** Institut Pasteur, Paris, France (C. Besombes, A. Gessain, N. Berthet, J.-C. Manuguerra, A. Fontanet);; Institut Pasteur de Bangui, Bangui, Central African Republic (E. Gonofio, X. Konamna, B. Selekon, E. Nakouné);; Centre National de la Recherche Scientifique, Paris (A. Gessain, N. Berthet);; Unité Pasteur-CNAM Risques Infectieux et Emergents, Conservatoire National des Arts et Métiers, Paris (A. Fontanet)

**Keywords:** Monkeypox virus, Orthopoxvirus, Central Africa, outbreak investigation, zoonoses, viruses

## Abstract

Monkeypox is a rare viral zoonotic disease; primary infections are reported from remote forest areas of Central and West Africa. We report an investigation of a monkeypox outbreak in Lobaye, southwest Central African Republic, in October 2018.

Monkeypox, a zoonotic disease caused by an *Orthopoxvirus*, has clinical signs and symptoms in humans similar to smallpox and a case-fatality rate of 10% (*1*). The specific reservoir species for monkeypox virus remains, to a large extent, unidentified (*2*). Spillover events of monkeypox have been reported in remote forest areas of Central and West Africa. After zoonotic infection, the virus can be transmitted from person to person (*1*).

To date, human monkeypox outbreaks in the Central African Republic (CAR) have been small: ≈10 cases, restricted to a family or village. Primary infection in these outbreaks occurred from contact with wild fauna, with secondary transmission among close contacts in the community (*3*,*4*) and limited nosocomial transmission (*5*). Since 2000, the Virology Laboratory of the Institut Pasteur de Bangui (IP Bangui), a regional reference center for monkeypox, has reported 20 monkeypox outbreaks across several regions of CAR, totaling ≈100 cases, particularly in the region of Lobaye (*3*,*4*). In 2018 alone, IP Bangui investigated 6 different outbreaks in CAR, indicating a possible increase in frequency of outbreaks *(**6*,*7*).

On September 27, 2018, a healthcare worker from Zomea Kaka healthcare center in Lobaye reported to IP Bangui about 3 cases of suspected monkeypox in an Aka Pygmy family. A 25-year-old female sought care at the health center, 10 km from her village, for maculopapular rash and lesions. She was afebrile. Her signs and symptoms indicated of resolving late stage monkeypox infection. She was accompanied by her 2 daughters, 5 months and 4 years of age, both showing typical symptoms of active monkeypox infection, notably maculopapular rash on the palms of their hands and soles of their feet (Appendix Figure). Blood or pus samples taken from the 3 patients were confirmed positive for monkeypox infection by PCR on September 29 (*8*) (Appendix).

On October 5, IP Bangui carried out an investigation among contacts of the index case-patient, in collaboration with the Ministry of Health and the World Health Organization CAR Country Office. The index case-patient reported butchering 3 small mammals known in local Aka language as Yabo (African civet, *Civettictis civetta*), Gbè (Emin’s pouched rat, *Cricetomys emini*), and Sende (African rope squirrel, *Funesciurus anerythrus*). She butchered 1 of each in a forested area 2 weeks before the onset of rash.

During October 6–10, two additional family contacts from the village, the index case-patient’s 2 sisters, 7 and 16 years of age, reported symptoms consistent with monkeypox infection. Healthcare workers collected blood or pus samples from the patients, and IP Bangui confirmed monkeypox infection by PCR. On October 26, monkeypox infection was confirmed in another family contact, the index case-patient’s 33-year-old sister-in-law. The dates of the onset of symptoms suggest 3 waves of intrafamilial transmission (Table) (*9*).

**Table:** Molecular and serologic evidence of index case and contacts with known and possible exposure to monkeypox virus, Central African Republic, 2018*

Patients	Age, y/sex	Symptom onset date	Signs/ symptoms	Animal contact	Collection date	Sample type			PCR‡			IgG§		Smallpox vaccine¶
						Blood	Pus†		MPXV	CPXV		MPXV	CPXV	
Index case-patient	25/F	2018 Sep 8	Rash, lesions	Y	2018 Sep 27	Y	N		+	–		–	–	N
Contacts														
Daughter	0.4/F	2018 Sep 20	Fever, rash, lesions	N	2018 Sep 27	N	Y		+	–		ND	ND	N
Daughter	4/F	2018 Sep 26	Fever, rash, lesions	N	2018 Sep 27	Y	N		+	–		–	–	N
Sister	16/F	2018 Oct 6	Rash, lesions	N	2018 Oct 8	N	Y		+	–		ND	ND	N
Sister	7/F	2018 Oct 9	Rash, lesions	N	2018 Oct 11	Y	N		+	–		–	–	N
SIL	33/F	2018 Oct 24	Rash, lesions	N	2018 Oct 25	Y	N		+	–		–	–	N
Mother	49/F	NA	None	N	2018 Oct 5	Y	N		ND	ND		+	+	Y
Son	13/M	NA	None	Y	2018 Oct 5	Y	N		ND	ND		–	–	N
Brother	49/M	NA	None	Y	2018 Oct 25	Y	N		ND	ND		+	–	Y
Brother of SIL	8/M	NA	None	NK	2018 Oct 25	Y	N		ND	ND		+	+	N
Nephew of SIL	13/M	NA	None	NK	2018 Oct 25	Y	N		ND	ND		–	–	N
HCW	34/M	NA	None	N	2018 Oct 5	Y	N		ND	ND		+	+	N
HCW	45/F	NA	None	N	2018 Oct 5	Y	N		ND	ND		+	+	Y
Social contact	22/F	NA	None	NK	2018 Oct 25	Y	N		ND	ND		+	–	N

*A total of 33 contacts were tested, 2 HCWs and 31 village contacts. CPXV, cowpox virus; HCW, healthcare worker; MPXV, monkeypox virus; NA, not applicable; ND, not done; NK, not known; SIL, sister-in-law; +, positive; –, negative. †Samples obtained by HCWs after training on collecting swab samples. ‡Quantitative and conventional PCR were performed by using generic primers G2R-G and Congo Basin primers C3L (*8*). §In-house tests were performed by using MPXV antigen isolated from local human cases and CPXV antigen related to Brighton Red strain. ¶History of smallpox vaccination was determined by verbal report and presence of scar.

IP Bangui conducted further investigations by using *Orthopoxvirus* serologic assays (Appendix) on blood samples collected from 2 healthcare worker contacts on October 5 and from 31 village contacts on October 25. Results revealed evidence of *Orthopoxvirus* serologic response in the index case-patient’s mother; 2 healthcare workers who had cared for the index case-patient; and the index case-patient’s brother, who brought her the wild animals (Table).

Serologic evidence of possible monkeypox infection can indicate prior exposure to the virus or, among persons >38 years of age, immunization against smallpox, and might explain the restricted size of the outbreak in the village. However, smallpox vaccination campaigns with a live-attenuated vaccinia virus ended in 1979 in CAR. Consequently, an increasingly larger proportion of the population is immunologically naive to *Orthopoxvirus* infection.

This investigation identified 5 clinical cases of secondary monkeypox infection spread over 3 waves of intrafamilial infection, originating from an index case-patient with primary infection possibly attributable to contact with wild fauna. The prompt declaration and isolation of suspected cases, as well as possible naturally acquired immunity or persistence of vaccine-derived immunity within the community, likely contributed to the restricted extent of secondary transmission. Further studies are needed to clarify risk factors for primary and secondary monkeypox transmission.

Positive serologic findings in healthcare workers during this investigation also highlight the limited infection prevention and control resources, such as isolation rooms, gowns, gloves, N95 respirators, and goggles, to protect healthcare workers responding to outbreaks in CAR. For communities located in remote forest areas in which zoonotic spillover and secondary transmission are thought to occur regularly, health center capacity and resources need to be strengthened. Health centers urgently need training on case recognition for healthcare workers, access to diagnostic capacities, and appropriate infection prevention and control measures to reduce the possibility of secondary transmission in these areas (*10*).

Appendix Additional information on intrafamily transmission of monkeypox virus, Central African Republic, 2018.
