# Supplementary material for: Intrafamily Transmission of Monkeypox Virus, Central African Republic, 2018
Source: Emerg Infect Dis. 2019 Aug;25(8):1602–4. doi: 10.3201/eid2508.190112 (PMC6649310; doi:10.3201/eid2508.190112)
Supplement: Appendix — Additional information on intrafamily transmission of monkeypox virus, Central African Republic, 2018. [file 19-0112-Techapp-s1.pdf]

Article DOI: https://doi.org/10.3201/eid2508.190112

Intrafamily Transmission of Monkeypox Virus, Central African Republic, 2018

Appendix

Methods for Diagnosing of Monkeypox Infection

The preferred method for diagnosing monkeypox infection in active cases is based on PCR detection of the virus on swabs or crusts of lesions (*1*,*2*). Blood samples can be used, the viremia starts with fever during the prodromic phase and lasting for an unknown duration. In this outbreak investigation, we used a quantitative and conventional PCR targeting the hemagglutinin gene and part of the A-type inclusion body gene, using generic and Congo Basin primers (*3*). This test has been used in previous outbreak investigations in CAR, contributing to several publications (*4*,*5*).

For the contacts, in the absence of clinical symptoms, we used in-house ELISA serologic assays for cowpox and monkeypox viruses. The cowpox virus assay used antigens from a Brighton Red strain (201, TCID50 $10^{6.5}$, prepared on MRC5 cell), and the monkeypox virus assay used antigens from a local strain (16/004) obtained during a previous outbreak (Bakouma region, 2016) in CAR. No formal validation of these serologic assays, known for their cross-reactivity among *Orthopoxviruses*, has been conducted.

Availability and logistics of diagnostic assays are a challenge in CAR and could result in incomplete screening and outbreak reporting in remote regions, with limited health and transportation infrastructure, or absence of specimen collection supplies. When cases are reported, IP Bangui organizes the primary care of patients, as well as sample collection and transport for testing in Bangui. Because crusts and vesicle swabs, or blood from patients with fever, are the preferred specimens for virus detection, only samples from symptomatic patients are tested by PCR. Samples from asymptomatic contacts are only tested with serologic methods.

References

1. Durski KN, McCollum AM, Nakazawa Y, Petersen BW, Reynolds MG, Briand S, et al. Emergence of monkeypox–West and Central Africa, 1970–2017. MMWR Morb Mortal Wkly Rep. 2018;67:306–10. PubMed http://dx.doi.org/10.15585/mmwr.mm6710a5

2. Di Giulio DB, Eckburg PB. Human monkeypox: an emerging zoonosis. Lancet Infect Dis. 2004;4:15–25. PubMed http://dx.doi.org/10.1016/S1473-3099(03)00856-9

3. Li Y, Zhao H, Wilkins K, Hughes C, Damon IK. Real-time PCR assays for the specific detection of monkeypox virus West African and Congo Basin strain DNA. J Virol Methods. 2010;169:223–7. PubMed http://dx.doi.org/10.1016/j.jviromet.2010.07.012

4. Kalthan E, Tenguere J, Ndjapou SG, Koyazengbe TA, Mbomba J, Marada RM, et al. Investigation of an outbreak of monkeypox in an area occupied by armed groups, Central African Republic. Med Mal Infect. 2018;48:263–8. PubMed http://dx.doi.org/10.1016/j.medmal.2018.02.010

5. Berthet N, Nakouné E, Whist E, Selekon B, Burguière AM, Manuguerra JC, et al. Maculopapular lesions in the Central African Republic. Lancet. 2011;378:1354. PubMed http://dx.doi.org/10.1016/S0140-6736(11)61142-2

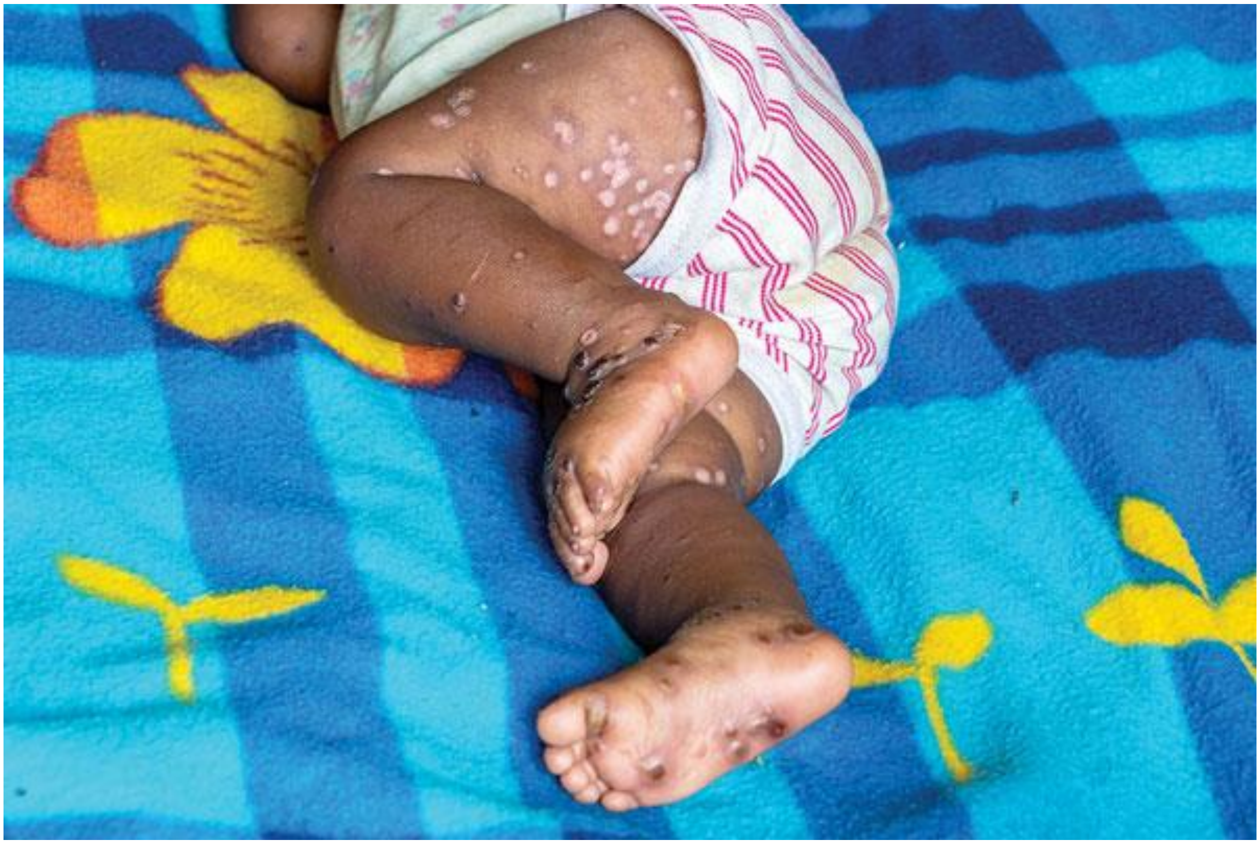

Appendix Figure. Image of maculopapular rash on 5-month-old daughter of an index case of monkeypox in Central African Republic, 2018.
